# Non-Invasive Physiological Monitoring for Physical Exertion and Fatigue Assessment in Military Personnel: A Systematic Review

**DOI:** 10.3390/ijerph18168815

**Published:** 2021-08-20

**Authors:** Denisse Bustos, Joana C. Guedes, Mário P. Vaz, Eduardo Pombo, Ricardo J. Fernandes, José Torres Costa, João Santos Baptista

**Affiliations:** 1 Associated Laboratory for Energy, Transports and Aeronautics, (LAETA/PROA), Faculty of Engineering, University of Porto, 4200-465 Porto, Portugal; ldbs@fe.up.pt (D.B.); jccg@fe.up.pt (J.C.G.); gmavaz@fe.up.pt (M.P.V.); 2 Porto Biomechanics Laboratory, University of Porto, 4200-450 Porto, Portugal; ricfer@fade.up.pt; 3 Commando Regiment, Portuguese Army, Serra da Carregueira, 2605-045 Sintra, Portugal; pombo.emv@exercito.pt; 4 Center of Research, Education, Innovation and Intervention in Sport, Faculty of Sport, University of Porto, 4200-450 Porto, Portugal; 5 Associated Laboratory for Energy, Transports and Aeronautics, (LAETA/PROA), Faculty of Medicine, University of Porto, 4200-319 Porto, Portugal; zecatoco@sapo.pt

**Keywords:** military operations, physiological variables, stressors, non-invasive methods, occupational health

## Abstract

During operational activities, military personnel face extremely demanding circumstances, which when combined lead to severe fatigue, influencing both their well-being and performance. Physical exertion is the main condition leading to fatigue, and its continuous tracking would help prevent its effects. This review aimed to investigate the up-to-date progress on non-invasive physiological monitoring to evaluate situations of physical exertion as a pre-condition to fatigue in military populations, and determine the potential associations between physiological responses and fatigue, which can later result in decision-making indicators to prevent health-related consequences. Adhering to the PRISMA Statement, four databases (Scopus, Science Direct, Web of Science and PubMed) were used for a literature search based on combinations of keywords. The eligibility criteria focused on studies monitoring physiological variables through non-invasive objective measurements, with these measurements being developed in military field, combat, or training conditions. The review process led to the inclusion of 20 studies. The findings established the importance of multivariable assessments in a real-life context to accurately characterise the effects of military practices. A tendency for examining heart rate variables, thermal responses, and actigraphy measurements was also identified. The objectives and experimental protocols were diverse, but the effectiveness of non-invasive measurements in identifying the most fatigue-inducing periods was demonstrated. Nevertheless, no assessment system for standardised application was presented. Future work may include the development of assessment methods to translate physiological recordings into actionable information in real-time and mitigate the effects of fatigue on soldiers’ performance accurately.

## 1. Introduction

Fatigue is a multifaceted phenomenon resulting from various factors. Several definitions of fatigue exist, usually based on the experimental protocol applied or the situation in which it occurs [1]. Generally, it can be understood as a condition of diminished individual capability to complete actions at the expected level due to lassitude or exhaustion of mental and physical strength [2,3]. From an occupational perspective, work-related fatigue is described as a mental, local or general non-pathological manifestation of excessive strain, entirely reversible with rest [4]. Furthermore, it has been associated with reduced human performance capability due to the inability to cope with physiological stressors [5].

Fatigue degrades performance and health, causing errors, incidents and accidents in operational contexts. Technological progress has made 24-hours-a-day and 7-days-a-week operations possible, integrating human activity worldwide and increasing exposure to fatigue-creating factors [6]. Military operations are fraught with such events, since the new technological complexity, the lethality of weapons systems, and the rapid worldwide response capabilities make soldiers’ performance more critical to mission success than ever before. The short- and long-term health of soldiers as individuals is also potentially at risk from military technologies that can surpass operator capabilities and compromise their safety [7,8].

Physical combat demands impose unique stresses on soldiers not seen with any civilian occupation [9]. They undertake land-based operations in harsh locations, involving high-altitude terrain [10], deserts [11,12], the tropics [13,14], and sub-zero temperatures [15]. Their most arduous duties are usually superimposed on multiple psychological and physiological stressors. These involve extended periods of near-continuous physical activity (frequently with load carriage above 30 kg [16,17]), impaired nutritional status and sleep deprivation [18], while maintaining high levels of alertness, executive function and, ultimately, job performance [19]. The combination of all these stressors can lead to severe physiological alterations and decrease physical and military performance on the combat field [5,20].

As stressors affect well-being and performance, optimising the efficiency of human activities involves developing methods to maintain both in the face of these issues. The early detection of effects of these stressors must be the premise when developing these methods. In this regard, physical exertion is the immediate result of a strenuous effort or use of energy that can later develop into physical and acute fatigue. As the literature evidences, physical exertion is the primary source of physical fatigue [21]. It alters the sympathetic nervous response, modifies oxygen uptake, and increases heart rate and blood lactate concentrations [22]. In other words, it activates a physiological adaptation to respond to the strenuous effort. As a result, various physiological variables have been used to assess physical exertion during physically demanding activities as they are objective indicators of physical endurance (when the body is able to cope with the physical stress) or conditions that can later develop into fatigue or severe health impairments [21,23,24].

The assessment of physical exertion through non-invasive physiological monitoring is described in the literature as a possible way to track the levels of effort involved during exhausting activities [21,25]. These levels of effort can be observed through upper and lower physiological limit values, which can also delineate the individual’s threshold limits above which their safety would be compromised. The development of physical fatigue can be seen as a process. It combines multiple contributors (personal, environmental, and operational demands) into a single physiological response [26,27]. Accordingly, it can be assessed by the continuous monitoring of the physiological variables: determining the pre-conditions of fatigue through physical exertion and predicting physiological overload or bad health status when reaching physiological limits.

Physiological data modelling determining individuals’ limits and the influence of moderating factors offers the basis for developing action plans that ultimately include how personnel eat, rest, train or are equipped. Therefore, it is essential to apply models that consider various stressors because soldiers are hardly exposed to only one stressor at a time [7]. In addition, investigations conducted in real-life stressful situations are crucial to expanding knowledge about the consequences of regular stress exposure, enabling the development of operationally useful approaches and improved treatments since they diverge significantly from controlled locations in terms of the environment, activity, equipment, and subject motivation [28].

Collecting physiological information in battlefield settings has repeatedly been obstructed by lack of access to in-theatre soldiers and methodological problems. However, thanks to the advances of non-invasive physiological monitoring systems, such information can now be recorded in military events. Accordingly, progresses in research within military populations have increased over the years [29,30,31,32], and reviews addressing their relevance have also been developed [5,16,33,34]. Consequently, it has become fundamental to understand whether investigation into non-invasive physiological monitoring has been conducted adequately in the military context, and if its results lead to successful physical fatigue assessments producing useful information.

Thus, the current review aims to (i) evaluate the up-to-date research on non-invasive physiological variables that have the potential to assess physical exertion and fatigue in real-life military settings; (ii) summarise the most-used methods to measure each of the identified physiological variables; (iii) determine the potential relationships between these variables, reference assessment procedures and subjective fatigue methods, and (iv) establish the current and future trends in the continuous measurement of physiological variables within military groups that can result in decision-making indicators to ensure personnel’s safety and health in an operational context.

## 2. Methods

This review followed the Preferred Reporting Items for Systematic Reviews and Meta-Analyses (PRISMA) statement [35]. In this regard, based on the related methodology [36,37], a protocol registered in PROSPERO (code CRD42018105833) was elaborated prospectively to determine adequate procedures to develop the review [38].

As indicated in the protocol [38], the research was conducted in three phases. First, through electronic databases, by using relevant search terms, combining two groups of keywords related to fatigue assessment and military performance. The first group with the words “fatigue”, “physical exertion” and “monitoring”, and the second with “military training”, “military performance” and “military operations”. The research was performed in the Scopus, Science Direct, Web of Science and PubMed databases. Each combination was identified in the title, abstract and keywords for the Scopus and Science Direct databases. The title and abstract were selected as the fields of search for PubMed, and the title was retrieved in Web of Science. Later, the process continued with backwards citation searching following the snowballing technique [39]. This procedure was repeated in the newly identified articles until no more relevant outcomes were obtained. Finally, additional sources found in citations were accessed.

The study selection was based on three phases of exclusion. Initially, filters were applied to the databases: (1) date—the results were limited to articles and articles in press from 2013 to 2020; (2) type of document—only research articles were considered; (3) type of source—items from peer-reviewed journals were selected, and (4) language—only publications in the English language were included. These criteria were adopted to assure the quality and relevance of the retrieved information. However, date restrictions were not applied when tracking the references of the first-obtained results. Then, the second and third phases were conducted by eliminating repeated records and analysing articles individually to remove those in which only subjective evaluation methods or unrelated goals were detected (reviews, case studies, and controlled laboratory trials).

The research focused on healthy, active military personnel with no age limits. Inclusion criteria for the final group were studies in which continuous physiological monitoring through non-invasive measurements was objectively performed, with these measurements being developed in military field, combat, or training conditions. At this stage, reasons for the removal of any record were respectively registered. From the finally selected studies, full texts were retrieved in order to collect information of interest using a customised table. The summary of information mainly included the following: reference, country, and keywords; sample size, gender distribution and mean age range; study goals, context, duration, conclusions, measured variables and equipment. The search and two first phases of the study selection were conducted by one author and confirmed by a second. The final phase for selecting studies and data to be extracted into the customised table was carried out by three independent researchers and verified by a fourth.

Finally, to determine the effectiveness of the monitoring procedures, the included articles were individually assessed for risk of bias. First, the general characteristics of each study were identified and analysed following the goals of the review. The factors considered included aims and objectives, assessed variables, applied methods and equipment, assessment procedure, and measurement time. Later, a customised table was applied to assess their risk of bias and methodological quality. This table was based on the Cochrane collaboration tool [40] and additional sources from the literature [41,42,43,44]. Within this table, each record was assessed individually through 25 previously established items divided into seven categories: study design, participants, data sources, reporting bias, limitations, generalisability, and potential sources of additional bias. Criteria were determined to address methodological difficulties and potential risks of bias in the obtained results.

The “study design” comprised seven questions on the rationale and clearness of the defined objectives and outcome measures, the existence of a control group, the research design, a description of methods and equipment, and the reporting of statistical analysis. The “participants” category (determined by five questions) verified if ethical standards were met and if the sample size was justified, and a randomisation method performed. It determined whether groups of subjects were similar at baseline regarding the most significant indicators, and whether the number of subjects was representative, in order to assure the study’s statistical power. Regarding the “data sources”, two criteria aimed to check the operational or training conditions of the experimental protocol and assure all subjects completed all testing.

Correspondingly, six questions in the “reporting bias” section determined whether the sample characteristics were adequately provided, and withdrawals and dropouts were registered. It also verified whether the available literature supported numerical data, whether complete outcome data were provided, whether tables and figures were clearly presented, and whether the data supported the authors’ conclusions. Two additional criteria were added in the “limitations” and “generalisability” categories determining whether limitations were objectively defined and whether the study allowed the generalisation of results. Finally, “potential sources of bias” (determined by three questions) identified additional causes of bias related to the withdrawal or dropout rate of participants, number of missing outcomes, and practical difficulties.

Each item was marked with Y (yes), N (no) or U (unclear) for the cases in which there was not enough information to define whether the criteria had been met. Then, rates were calculated by averaging the number of positive answers from each category and adding up the results. Studies presenting higher scores on the established criteria were considered as the most suitable and reliable for the objectives of this review. Conversely, investigations with a higher percentage of negative answers would immediately be considered for exclusion. A meta-analysis was not performed because of the differences in study protocols (recordings duration, assessed variables, military personnel characteristics, military activities and environmental conditions) and the lack of a comparator. Thus, outcomes were tabulated and described narratively. This research was performed after publication of the protocol in 2018 and updated in 2020.

## 3. Results

### 3.1. Articles Selection

Following the PRISMA statement guidelines [35], 6453 items were initially retrieved from the database searches. Then, using the search engines’ filters, date, article type, source type, and language restrictions were applied. Details concerning the number of rejected articles with each filter are presented in Table 1. After concluding this phase, 1307 articles were identified, of which 405 were duplicates, leaving 902 items for a third phase. During this last stage, 858 articles were excluded for not being applied within a sample of soldiers or involving only subjective measurement methods, and 44 articles were left for a final full-text assessment.

**Figure 1:**
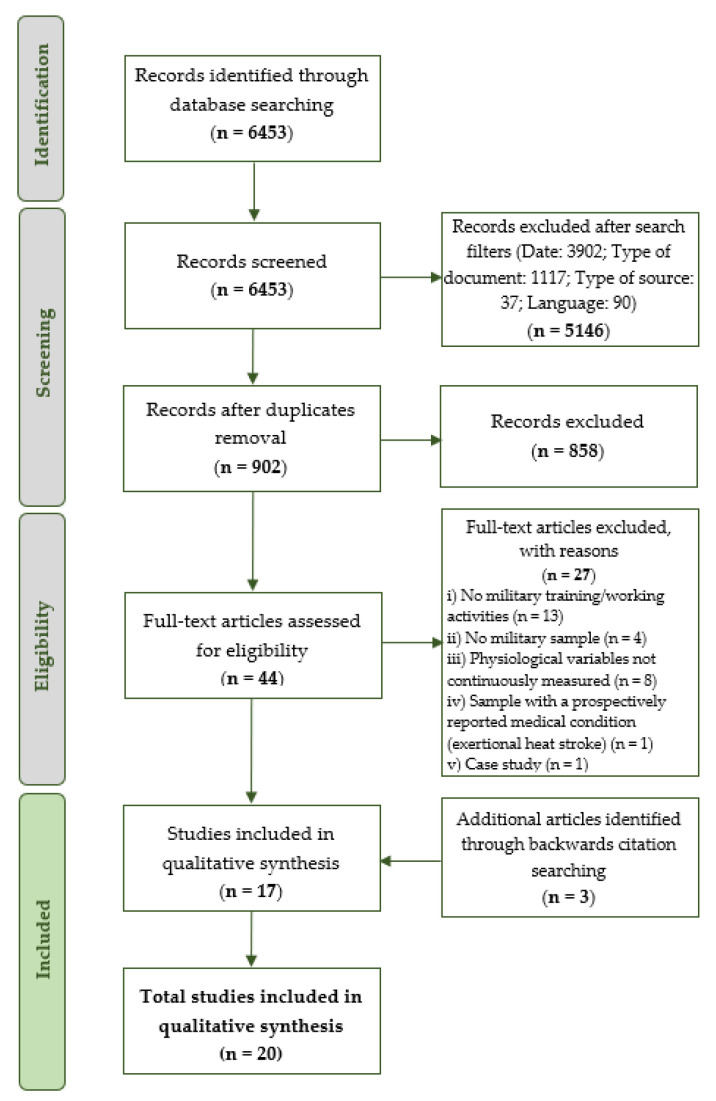
Summary of the research, based on PRISMA flow diagram [35] and protocol [38].

In this next phase, each manuscript was analysed to determine its accordance with the eligibility criteria. As a result, 13 articles were excluded for not involving any military training or operational conditions. Four studies were eliminated for not including a sample of regular military elements. Eight studies did not include any continuous measurement of physiological variables. Finally, two items were filtered for not involving healthy subjects and being identified as a case study. Following this process, it was possible to identify 17 relevant publications. A further three studies were obtained from backward reference searching of the 17 included studies. Therefore, 20 articles met the inclusion criteria and were selected for the systematic review. Figure 1 provides an overview of the number of articles identified at each stage delimited by the PRISMA methodology [35].

### 3.2. Overall Findings

The included articles are presented in Table 2, summarising the study objective, duration and assessed variables. The studies included 2589 individuals, of which approximately 2513 were men and 76 women, representing 97% and 3%, respectively. All participants were healthy, active military personnel, and their ages ranged from 20.1 to 34.5 years. The duration of the studies varied widely, ranging from one day of a recruit training course [45] to six months of an international crisis management operation [46]. Two studies included a control group of soldiers who were not in training and were monitored while carrying out regular military duties [47,48]. In contrast, the rest used comparisons with subjects’ basal or previous levels. This review focused on physiological monitoring during regular in-field, combat or training activities. Only regular baseline conditions were considered for analysis from the two studies addressing interventions [47,48].

Of the 20 manuscripts, 16 described experimental moments that occurred during different training activities (mountain warfare cold-weather training [64]; chemical, biological, radiological, nuclear and explosive materials defence [58]; combat [50,51,53]; captivity survival [59,60]; usual military practices [45,49,55,56,57,61,62,63]). Two were applied during operations [46,54]. Two others involved both scenarios [47,48]. Furthermore, considering continuously assessed variables through non-invasive methods, most of the papers (*n* = 15) examined different heart rate-related variables [45,46,47,48,49,50,51,54,55,56,57,58,61,62,64] while 10 monitored variables related to the estimation of physical activity mainly based on accelerometer counts [46,50,51,52,53,54,56,57,60,63]. Three studies were also found that assessed thermal responses, two recording core temperature measurements [54,58] and one evaluating body skin temperature [57]. This latter investigation included a continuous recording of the breath frequency [57], along with both heart rate and physical activity variables.

As most of the studies performed heart rate measurements [45,46,47,48,49,50,51,54,55,56,57,58,61,62,64], significant increases in mean heart rate values during the most demanding stages of the applied protocols were a consistent finding among the retrieved papers. Due to the heterogeneity of outcome measures within this review, a comparison of obtained values could not be performed. Still, some remarkable results could be identified. Increases of up to 81% from baseline were observed in the absolute values of heart rate during mock interrogations in captivity survival training [59], and 64% increases in %HRR (calculated by subtracting resting HR from the exercise HR, dividing by the difference between resting and maximal heart rate, and multiplying by 100% [65]) were evidenced during load carriage tasks. A 70% increase in the same variable was observed in uphill walking [45].

Considering physical activity recordings, eight studies used accelerometers to collect sleep, rest or activity periods [46,50,51,52,53,56,60,63]. Two of them simultaneously used physical activity trackers (a physical activity tracking software installed on smartphone devices) and logs [52,53]. The two additional studies addressing activity counts retrieved information from an Equivital monitoring system [54,57]. In general, data obtained accurately characterised the intensity of activities performed. However, the most significant outcomes were derived during the observation of sleep periods. They evidenced a night rest of approximately 5 h [50,60] and a later decrease of up to 1 h and 45 min after exposure to the acute and severe stress associated with military practices [60]. Regarding thermal responses, skin temperature was successfully recorded with the Equivital system [57]. Two studies [54,58] used thermometer pills to monitor core temperature responses. In general, outcomes from this variable evidenced increases in thermal responses directly proportional to the stress of military training.

Finally, concerning the processing and analysis of physiological variables, traditional methods were the clear tendency. Monitoring records were gathered and analysed retrospectively, and most of the articles referred to statistical tests to examine the effects of training events and present their outcomes. Limited evidence was derived on the use of other processing approaches, as only one study was found that developed an estimation algorithm for core temperature from heart rate recordings [58]. Among the studies, no real-time assessment methods were retrieved.

### 3.3. Risk of Bias and Quality of Results

Using the Cochrane collaboration tool as a reference [40], methodological issues were addressed (fulfilling ethical standards, sample justification, clear descriptions of the experimental procedure, and practical difficulties). A customised table was applied to examine their potential risk of bias and methodological quality. As previously mentioned, the included articles were assessed through the 25 established criteria referring to study design, participants, data sources, reporting bias, limitations, generalisability, and potential sources of additional bias.

In general, all studies were positively rated in methodological quality since they presented more affirmative answers to the proposed criteria (scores above 3.5). However, none reached the highest possible score, suggesting that bias was present in all of them in smaller or larger proportions. Details of methodological weaknesses are compiled in Table 3 and explained in Appendix A. Most of the negative responses were observed when evaluating the randomisation procedures of samples (in the “participants” category) and the uniform completion of all parts of the proposed protocols (addressed in the “data sources” section). Methodologically, only three studies declared any form of randomisation procedures [56,59,61]. None indicated power adjustment or consideration to account for the correct sample size. The sample sizes ranged from 10 [54] to 1676 [56].

Most of the evaluated criteria were fulfilled regarding the studies’ design, and weaknesses were only found when comparing outcomes with control groups (only two studies reported them [47,48]). Concerning the “outcomes” category, a detailed description of withdrawals and dropouts was not present in most evaluated articles (12 out of 20). Finally, the “limitations” assessment revealed that nine out of the 20 studies did not describe the limitations and opportunities for improving their research.

### 3.4. Characteristics of the Included Studies

As observed in Table 2, the groups of variables identified in each article were diverse, and none of the studies specifically focused on fatigue assessment. They did, however, address different dimensions of training that can lead to both physical exertion and fatigue. Among them, some common assessment goals and related evaluated variables were retrieved (see Table 4). Most of the manuscripts were focused on determining the effects of specific training practices. In these, non-invasive measurements were combined with reference variables and provided corresponding results. Table 4 helps to describe the associations between variables (the objective physiological variables aimed in this review, the reference assessment methods and the subjective fatigue indicators) and lists the outcomes obtained from each study. In general, noninvasively collected measurements were accurate in characterising the most stressful periods of training. They showed associations with other variables with proven validity as stress markers, such as biochemical indicators.

In cases where no reference variables were identified and results were compared with perceived exertion or other subjective assessment methodologies, the outcomes still demonstrated accuracy in determining physical activities’ intensity and physiological and mental workload. In this regard, quality assessment scores (in descending order in Table 4), which were derived from the risk of bias assessment in the previous section, helped infer the quality and generalisability of the outcomes, and how methodological difficulties associated with real context monitoring may have influenced them. As a result, within the retrieved studies, the effectiveness of non-invasive monitoring in real contexts was verified.

## 4. Discussion

This study aimed to develop a comprehensive search of the literature to delimit the current progress in non-invasive physiological monitoring, which can lead to physical exertion and fatigue assessment methods within military operational settings. A total of 20 studies conducted under field or training conditions met the inclusion criteria. Their predominant focus was on quantifying the effects of specific military training programmes. The search identified that heart rate and its derived variables were the most evaluated responses. Accelerometer-estimated physical activity and thermal variables were also common among the assessments. An interest point in examining responses to heat strain was identified. A more recent focus on estimating energy expenditure from specific military activities was also observed. Finally, it was verified that decisive outcomes were not obtained within the same group of variables and, since the assessment procedures are also diverse, no standardised assessment methodologies were identified.

### 4.1. Monitoring in Field Context

Technological advances increasingly enable the real-time assessment of physiological variables. Devices are decreasing in size, improving in functionality, and becoming usable for military operational settings. The feasibility of obtaining data from real operational or training conditions to contextualise physiological variations accurately was demonstrated in the retrieved publications. However, evidence of the specific effects of sustained operations, the impacts of prolonged exposure to extreme environments, and changing mental and physical task demands were proven to be limited. The primary goals of the papers were mostly oriented to determining the overall effects of military practices. Experimental protocols and respective analyses were mainly dedicated to the short-term effects of these training or operational duties.

The findings reveal a clear tendency towards assessing the effects of different dimensions of training on performance. In total, 16 of the 20 manuscripts were oriented in this regard, while other two centred on comparing measurement methods. Notably, only two investigations aimed to validate the application of a monitoring assessment method and an estimation algorithm, respectively. This latter [58] obtained a similar overall performance of their proposed algorithm in operational settings when compared to a previous study developed in laboratory conditions. This outcome supports the assertion that, despite ambient limitations, reliable data can be obtained in operational circumstances. More significantly, it exemplifies the research opportunities derived from the optimisation of data treatment methods, such as the proposed algorithm.

Regarding the military practices evaluated, studies show how these real-life environments are essential to accurately evaluate soldiers’ responses to stress. As observed in Table 4, the captivity survival course referred to in Lieberman et al. [59] resulted in the simultaneous alteration of variables during the simulated captivity phase in all examined dimensions. Heart rate elevation agreed with increases in biochemical stress markers, and decrements in mood states degraded cognitive function. Additionally, significant body weight loss was observed in this phase. Remarkably, despite the differences in samples and programme duration (four days vs. three weeks), results from Ralph et al. [60] verified that physiological performance during the Conduct After Capture programme (which programme and phases (didactic, practical, and debriefing) resemble the main features of [59]) was significantly degraded with training, mainly during the most intense interrogation, comparable to what was determined by Lieberman et al. [59]. The correspondences of both protocols and responses suggest that, potentially, both monitoring results can be assessed by standardised systems able to deliver an overall view of the degraded performance of the soldiers.

Consequently, the outcomes point to both the need and the viability of conducting in-field studies to characterise the effects of these stressful practices accurately. The literature demonstrates how military service entails a host of stressors, generic and exclusive [5,66]. The degree to which information can be provided regarding what kind of responses under specific circumstances can be expected determines the ability to prevent these stressors later. Thus, continuous short- and long-term analysis should be performed. An immediate analysis is essential to explain how every stage of the training affects the subject [67]. Long-term analysis would also permit the identification of specific behaviours and anticipate further health impairments at the time of determining whether recovery and training periods are balanced [68]. In general, the manuscripts included in this review were focused on the first evaluation. The sustained examination of responses is suggested for future studies. Furthermore, concerning data treatment methods, a lack of existing knowledge on validated assessment tools to treat the results from these in-field studies was observed. Understanding the factors affecting military populations, standardised methods, and assessment tools monitoring these factors should be developed and validated in future research.

### 4.2. Physiological Information

Among studies, it was possible to identify non-invasive and straightforward measurements that could fit into the soldiers’ schedule. Cardiac responses to military training and missions were found as the most common focus among the papers retrieved. This focus is explained by the cost-effectiveness and practicality of available measurement procedures, as well as their proven applicability in almost any situation [69]. Regarding measuring methods, the studies mostly recorded heart rate through Polar heart rate monitors (Polar Electro, Kempele, Finland) and by using chest belt physiological monitoring systems (Equivital EQ-01 Hidalgo, Cambridge, United Kingdom). Polar heart rate monitors are lightweight telemetric monitors, recognised as the most accurate devices for heart rate monitoring and recording in the field [70,71]. Equivital monitors are integrated systems, able to measure not only cardiac but also thermal and breathing responses [72,73]. Both devices have validated reliability for high-intensity training scenarios and field conditions. This review showed their good applicability for military settings as well.

Heart rate is a useful non-invasive quantitative indicator of physiological adaptation and intensity of effort [74]. Correspondingly, the results reveal that heart rate and its derived variables were examined in 15 out of the 20 studies included (see Appendix B). Most of them focused on absolute values determined in beats per minute. In contrast, others expressed cardiac measurements as percentages of baseline values or maximal obtained or theoretical values. In all cases, this variable evidenced ups and downs directly proportional to the different intensities of training protocols. Scales to classify the obtained cardiac variations were also identified. However, they were diverse among the studies. Beals et al. [64] made reference to the three-level scale for physical intensity based on maximal heart rate percentage from Garber et al. [75]. For the same purpose, Pihlainen et al. [55] used Howley et al.’s [65] six categories, while Fallowfield et al. [45] suggested the same authors [65] for determining the level of cardiovascular strain with five heart rate reserve percentage categories. Finally, both O’Leary et al. [62] and Clemente-Suárez et al. [51] classified the responses of maximal heart rate percentage in five [76] and six zones [77], respectively. 

For real-time settings, these heart rate-based intensity scales have compelling advantages, as they are a simple approach to understanding the interactions between fatigue, physical exertion and performance compared to more complex and large physiological records. Since cardiac reactivity can differ considerably from one person to another, and baseline measurements can also be varied, the tendency to consider heart rate-derived variables such as heart rate variability, and percentages of maximal heart rate and heart rate reserve (instead of absolute values), was observed as the most feasible approach in the face of the variability within samples. 

The literature has evidenced how continuous methods of heart rate measurement are essential for preliminary diagnostic indicators of any form of cardiovascular risk (cardiac diseases or stroke) [78,79] resulting from intense, physically demanding situations. However, multiple factors can also influence increases in heart rate (stress, anxiety, body posture) without necessarily representing any health risk. The results revealed that, given the wide range of variability in subjects’ heart rates, this marker could not be used solely to assess fatigue or related conditions. In fact, in none of the 15 studies in which cardiac responses were included were they the only measured variable. Heart rate monitoring was mostly performed along with physical activity patterns and thermal responses. In addition, the results were generally compared with responses from subjective variables. Thus, it can be concluded that cardiac metrics are essential yet not definitive physical exertion and fatigue indicators. The combined assessment of these and thermal or accelerometry variables can be inferred as an alternative to reduce the potential biases resulting from their variability.

Variables related to quantifying physical activity patterns were the second most recurrent monitoring approach (see Appendix B). Wrist actigraphs were generally used [47,48,50,60]. Other methods, such as physical activity logs and direct observations using physical activity trackers, were also tested [52,53]. Although the results evidenced correspondences in the data obtained from the different methods, the authors concluded that accelerometry counts were the most suitable variable to determine the physical intensity of activities [52,53]. Physical activity trackers and physical activity logs were found to be better for reporting the type of physical activity and body position. Consistently, accelerometers have proven to be generally accepted as practical and useful sensors for wearable devices to record and evaluate physical activity patterns in laboratory settings and free-living environments [80]. Compared to the previously outlined cardiac metrics, data from accelerometers offer useful complementary but not interchangeable information. They give information on the characteristics and intensity of the activities, while heart rate helps determine the impact of those activities on the individual.

Collectively, the findings highlight the relevance of accelerometer-derived physical activity monitoring not only for evaluating activity periods along with posture and movement classification, fall detection, and assessment of energy expenditure, but also for measuring the sleep decrements caused by training. However, the literature has proven that combining heart rate or other heat-sensing technologies is required to decrease the error when classifying activity types [81] and accurately characterise the impact of training through energy expenditure estimation. Moreover, when intending to establish associations with stressors such as sleep deprivation, the inclusion of any cardiac metric appears essential to determining any cardiometabolic health relationship.

Concerning thermal responses, core temperature proved to be a valid marker of acute stress and physical intensity variations. Interestingly, all studies assessing core temperature (continuously or not) also reported heart rate measurements [47,48,49,54,58]. Consistently, normative guidelines outlined in the ISO 9886 (2004) [82] determined the relevance of correlating both variables. The limit values for normative ranges reach from 38 °C to 39 °C. However, within military populations, measurements reach above 39 °C [58,83,84], evidencing not only the unique physiological demands of military training, but also the need for additional markers to provide a definite diagnosis on the level of the heat strain and related physical exertion faced by soldiers. In accordance with normative criteria [82] and the available literature [85,86], core temperature should not be used as an isolated measure to evaluate real-time heat strain and the derived physical exertion, as the interaction among environment, physical demands, personal protective equipment, individual anthropometrics and fitness in the development of heat-related illness is multifaceted. It is a critical variable when assessing heat strain and preventing thermoregulatory disorders. However, it is only a guide, and not the endpoint of health assessments [87].

On the other hand, relatively little evidence was gathered on skin temperature recordings. This variable was included in three papers [48,49,57], in which heart rate and core temperature responses were also retrieved. Skin temperature was not continuously monitored in all cases. A correspondence between obtained skin temperature and perceived values of exertion was proven, and a potentially positive influence on work capacity caused by the conscious perception of the thermal environment was highlighted [48]. Although only three studies referred to this variable, they helped understand the potential of measuring temperature on specific body parts to detect physical exertion and fatigue development. Heat exhaustion is evidenced through high skin temperatures associated with peripheral blood flow, which decreases venous return, cardiac filling and stroke volume, causing cardiovascular strain [87]. The evolution from heat exhaustion to heat stroke may occur quickly. Thus, the continuous monitoring of both skin temperature and heart rate would permit a timely intervention to prevent it.

Regarding breathing rates and any other respiratory variables, they were measured by some multiparameter wearable devices identified in this review. However, of the selected studies, only one manuscript [57] referred to assessing the frequency of breath. The available literature suggests that frequency of breath can provide useful information regarding the overall physical condition of a subject [88,89,90]. Therefore, further studies can assess the feasibility of integrating this variable.

Concerning the assessments performed, analysis and data treatment methods were found to be limited. Up-to-date knowledge focused on the separate analysis of single physiological signals. Multivariable measurements were performed, but conclusions were drawn by gathering individual results from each variable. There was a lack of adequate classification methods, and no multivariable models existed that combined these signals in a reliable and valid way. As a result, the emerging concerns for future investigations include the validation and standardisation of monitoring methods and systems involving multiple variables, as well as data handling, algorithms, and multivariable models to treat the results from monitoring.

The vast amount of information derived from physiological sensors has to be structured, annotated, and should be made accessible. Within the studies included, it was demonstrated that physiological variables such as heart rate, thermal responses, and breathing reflect the psychophysiological body status resulting from physical exertion. Accelerometry allows for determining specific behaviour patterns and general activity levels. Combined data from both signals offer a much more complete image of a current bodily state. Information gathered from this review supports the need for assessment systems combining these variables. They will allow analyses and modelling techniques to be applied to reveal essential factors influencing military performance in terms of operational readiness and sustainability.

Traditionally, the combined evaluation of variables leads to more solid results. Examples of these integrated approaches are observed in the literature for different purposes, such as enhancing energy expenditure estimation [91] or assessing health conditions remotely [92]. Within occupational areas, they have been used to report life sign conditions among military groups [93], or even to address physical exertion modelling within construction workers [21]. In the military, the results from continuous monitoring may be used in early screening for symptoms of chronic stress and as the basis for further contact with medical care. This review contributes to the findings of preceding investigators in the study of physical exertion leading to fatigue in military populations. It can be used as a starting point for future investigations to examine what has been done, where further work is needed, and which are the points of departure for developing new research.

### 4.3. Current Trends and Future Perspectives

Several physiological variables have the potential for use in the real-time assessment of physical exertion and fatigue. The findings evidenced a trend of measuring heart rate, thermal responses and variables related to physical activity estimation. A multivariable assessment combining these might be the most appropriate approach for a complete characterisation of fatigue within military populations. Heart rate-related metrics are definitely the most commonly used physiological metrics to measure physical exertion, not only among analysed studies but also within other occupational groups (such as construction workers [94] and miners [95,96]) and sports-related fields [85,97]. Specifically, heart rate variability (HRV) has been reported as a valuable index of cardiovascular health and well-being that provides an insight into the physiological alterations resulting from work-related fatigue [98]. However, the multidimensional nature of fatigue and the various stressors to which soldiers are exposed suggests these metrics cannot provide a conclusive diagnosis of fatigue-preceding conditions. 

In this regard, the outcomes suggest that other metrics, such as body temperature (skin and core), breathing patterns and accelerometry counts from actigraphy, can also evaluate physical exertion leading to fatigue. Although these variables are promising for use in fatigue management models, the heterogeneity of the examined studies offers limited evidence regarding which combinations of these variables are the most suitable for better characterising physical exertion in military groups. However, the individual analysis of variables among these papers does report useful insights about the effectiveness of these variables in describing the most demanding periods of training. Thus, their integrated assessment must lead the way in future research.

Interestingly, the approach of combining these variables is consistent with previous studies reporting this as a good method of physical exertion monitoring and fatigue assessment, among other areas. For instance, studies from the construction field [99] have revealed the need for multiple physiological metrics (including heart rate and heart rate variability, skin temperature and activity estimation metrics) to more accurately detect fatigue. Several authors have proven the importance of cardiorespiratory and thermoregulatory variables for developing early-warning models for fatigue detection and continuous monitoring [21,94,100]. Occupations exposed to severe sleep deprivation along with other stressors (similar to some military populations) have confirmed the relevance of combining heart rate variability and accelerometry counts from actigraphy to determine the changes in fatigue levels [101]. 

Furthermore, the literature also shows that when combining physical demands with exposure to hot environments (as typically observed among the military), the risk of heat-related illnesses requires the monitoring of thermal responses to protect armed forces personnel against these health impairments. As a result, investigations from related areas show the validity of combining physiological metrics to accurately evaluate physical exertion leading to fatigue, and the results from this review evidence the feasibility and trend of measuring one or more of these variables, pointing to future perspectives in which their integrated and long-term assessment approach appears to be the most solid direction.

### 4.4. Limitations

It should be stated that, in the selected studies, some limitations were identified, mostly associated with the expected difficulties of measurement in field conditions. Many of the studies described differences in the numbers of soldiers completing each assessment, and others reported that not all participants finished all phases of the training events. A comprehensive reporting of experimental protocols is suggested to improve the validity of future investigations, including details on the inclusion and exclusion criteria, baseline demographics, and handling withdrawals. In most, if more volunteers had participated, more robust effects may have been detected.

Furthermore, as was reported by some authors, it was not always feasible to continuously record all variables, which would have allowed better analyses of results. Besides this, only two studies reported a control group, and comparisons with groups not taking part in the practices would have permitted the detection of any possible baseline bias. In addition, the described technical challenges (noise and signal artefacts, user acceptance) compromised the validity of recordings and led to the elimination of data from some parts of the training. These issues need to be addressed in upcoming research to achieve effective real-time fatigue monitoring. Finally, the samples were predominantly males. Even though this characterises what is normally seen in military groups, caution must be exercised when drawing inferences and generalising to both men and women.

Concerning the limitations from this review, publication bias was observed as one limitation. Within this work, no unpublished research was included. In addition, language restrictions when filtering items may have left out some relevant studies. Another potential limitation pertains to the differences in sample sizes and the group of variables assessed across the studies, which interfered with a more in-depth assessment of all of them. Moreover, the customised scale used to determine the risk of bias in the included articles may not have been the most suitable for all studies, given their heterogeneity.

## 5. Conclusions

The data from this systematic review process compile evidence of the current progress on non-invasive physiological monitoring approaches that can be used for physical fatigue assessment within military personnel engaged in training or operations. From it, some final considerations can be highlighted. First, considering the multidimensional nature of fatigue, it was demonstrated that its assessment cannot be performed with one or two variables. The importance of measuring multiple variables is emphasised for a complete characterisation of its influence on physical and physiological functioning. Heart rate variables proved to be the most commonly used to assess physical exertion and various fatigue-related factors, but other variables such as thermal responses, respiratory rate and movement patterns from accelerometry were also useful in this regard.

Second, the results demonstrate that military training events such as those evaluated throughout the studies produce high levels of stress to simulate combat, providing an excellent opportunity to assess the physiological responses of humans to fatigue. Overall, within the publications included, it was proven that valid measurements could successfully be recorded from these events, paving the way for individualised fatigue assessment in real-time and the continuous tracking of soldiers’ well-being. Future perspectives should be encouraged to use these continuous assessment procedures to help anticipate illnesses or injuries and improve work or training schedule management.

Finally, referring to the current trends in processing and assessing physiological recordings, the outcomes revealed that evidence of physical exertion and fatigue assessment procedures is limited regarding innovation and real-time methods combining different variables. There is a lack of standardised methods or systems for examining the obtained measurements and providing an overall interpretation of the reached limit values, which could lead to timely interventions and prevent health-related adverse effects. Therefore, future works must be oriented towards developing these systems with applicability to military personnel or any safety-sensitive profession.

## Figures and Tables

**Table 1:** Summary of the first phase of the screening stage based on protocol [38].

Database	Total Number of Identified Articles	Summary of Rejected Articles				Total Number of Selected Articles
		Date	Type of Document	Type of Source	Language	
Scopus	5929	3650	1071	37	87	1084
PubMed	120	64	0	0	2	54
ScienceDirect	380	174	44	0	1	161
Web of Science	24	14	2	0	0	8
**Total**	**6453**	**3902**	**1117**	**37**	**90**	**1307**

**Table 2:** Characteristics of selected studies (ordered by publication year) based on protocol [38].

Study/ Country	Study Objective	Study Design (Baseline/Intervention)	Duration	Non-Invasive Physiological Variables	Other Monitored Variables
[49]/Australia	To examine physiological and psychological responses to heat strain during operations in the tropics	Baseline comparison	3 days	Heart rate, rectal temperature *, skin temperature (thigh position) *	Environmental conditions; oxygen consumption, water consumption, and fluid balance; cognitive and psychological performance; weight loss
[50]/USA	To examine cognitive and physiologic effects of brief but intense stressors	Baseline comparison	5 days of a three-phase training	Accelerometer-based physical activity (estimated duration, sleep hours)	Cognitive performance; mood, vigor, fatigue, confusion, depression, and tension; cortisol, testosterone, and melatonin; body composition and hydration status
[45]/UK	To quantify the cardiovascular strain of a load carriage event	Baseline comparison	1 day (270 min)	Heart rate	Body composition, maximal aerobic capacity, estimated oxygen consumption; speed of movement, altitude, and distance covered; neuromuscular performance and effects of load carriage; environmental conditions
[51]/Spain	To examine physical, mechanical, and physiological responses to prove that symmetrical and asymmetrical combats are different	Baseline comparison	2 days	Heart rate, Accelerometer-based physical activity	Speed, sprints, distances, impact, body load parameters; body composition; environmental conditions
[52]/USA	To compare 3 methods for measuring physical activity	Baseline comparison	8 weeks	Accelerometer-based physical activity	No additional variables
[53]/USA	To compare physical activity variables in two training sites	Baseline comparison	4 months	Accelerometer-based physical activity	No additional variables
[54]/USA	To quantify thermal work strain and predict its effects during another mission	Baseline comparison	2 months	Heart rate, core temperature, physical activity based on accelerometry counts	Meteorological data: air temperature, dew point and black globe temperature; clothing insulation and vapour permeability; height, body weight, waist circumference at the navel, fighting weight; metabolic rate and body fat
[55]/Finland	To examine cardiorespiratory responses during military tasks with loads	Baseline comparison	20 days	Heart rate, respiratory patterns *	Rates of perceived exertion; distances moved, altitude differences and velocities; body composition; oxygen consumption
[56]/Switzerland	To investigate the impact of training on injury incidences	Baseline comparison	18 weeks	Heart rate, physical activity-related variables (hip acceleration counts, step counts)	Physical activity energy expenditure, distance covered on foot; time spent on physically demanding material handling activities, sport-related PT, inactivity night rest; monotony and development in weekly training load variables; injury log and training reports
[57]/The Netherlands	To implement a monitoring assessment system and to establish a set of determinants that best predict attrition in infantry training	Baseline comparison	24 weeks	Heart rate, frequency of breath, skin body temperature, acceleration counts	Cognitive performance, anthropometric measurements, somatotyping, psychological determinants
[58]/USA	To evaluate the performance of a core temperature estimation algorithm	Baseline comparison	6 days	Heart rate, core temperature	No additional variables
[59]/USA	To assess cognitive, affective, hormonal, and heart-rate responses to survival training	Baseline comparison	3 weeks	Heart rate	Biochemical stress markers in blood and saliva; cognitive performance, mood states
[47]/France	To assess the effects of adding a 5-day acclimatisation training programme before mission	Intervention (control group)	7 days	Heart rate, rectal temperature *	Sweat loss and osmolality, thermal discomfort, weight, environmental conditions
[60]/Canada	To examine effects of captivity survival training	Baseline comparison	4 days	Activity, sleep, and rest periods based on actigraphy data	Mood, fatigue, PTSD symptoms, dissociation, short-term memory and working memory; biochemical stress markers in blood and saliva
[61]/Italy	To compare energy expenditure equations and heart rate-based estimates of army loaded runs	Baseline comparison	6 months	Heart rate	Estimated energy expenditure, environmental conditions, body weight, mean speed and distance
[48]/France	To assess the effects of adding a 15-day acclimatisation training programme before a mission	Intervention (control group)	17 days	Heart rate, rectal temperature *, facial skin temperature *	Osmolality, sweat loss, thermal discomfort; rates of perceived exertion; environmental conditions
[62]/UK	To determine sex differences in training loads during basic training	Baseline comparison	14 weeks	Heart rate	Distance, training impulse; rates of perceived exertion; energy expenditure
[46]/Finland	To investigate changes in physiological and biochemical markers during military management operation	Baseline comparison	6 months	Heart rate and physical activity counts from accelerometry	Average ambient temperature; biochemical stress markers in blood and saliva; rates of perceived exertion; body composition
[63]/Czech Republic	To determine if energy balance remained steady during training	Baseline comparison	1 week	Physical activity based on actigraphy	Energy expenditure, energy intake
[64]/USA	To examine energy demands of training to improve fueling conditions	Baseline comparison	3 days	Heart rate	Energy intake, body composition changes, aerobic capacity, lactate threshold

* Variables not continuously measured.

**Table 3:** Methodological assessment of selected studies and risk of bias analysis.

Study	Study Design	Participants	Data Sources	Reporting Bias	Limitations	Generalisability	Potential Other Sources of Bias	Score (0–7):
[49]	0.86	0.40	1.00	0.83	0.00	1.00	1.00	**5.09**
[50]	0.86	0.80	0.50	0.83	0.00	1.00	1.00	**4.99**
[45]	0.86	0.60	1.00	1.00	1.00	1.00	1.00	**6.46**
[51]	0.86	0.80	0.50	0.83	0.00	1.00	1.00	**4.99**
[52]	0.86	0.80	0.50	0.67	0.00	1.00	1.00	**4.82**
[53]	0.86	0.80	0.50	0.67	1.00	1.00	1.00	**5.82**
[54]	0.86	0.20	0.50	1.00	1.00	1.00	0.67	**5.22**
[55]	0.86	0.40	0.50	1.00	1.00	0.00	0.33	**4.09**
[56]	0.86	1.00	0.50	0.83	1.00	1.00	1.00	**6.19**
[57]	0.86	0.80	0.50	1.00	0.00	1.00	1.00	**5.16**
[58]	0.86	0.40	0.50	0.83	1.00	1.00	1.00	**5.59**
[59]	0.86	1.00	0.50	1.00	1.00	1.00	1.00	**6.36**
[47]	1.00	0.80	0.50	1.00	1.00	1.00	1.00	**6.30**
[60]	0.86	0.80	0.50	1.00	1.00	1.00	1.00	**6.16**
[61]	0.86	1.00	1.00	0.83	1.00	1.00	1.00	**6.69**
[48]	1.00	0.80	0.50	1.00	1.00	1.00	1.00	**6.30**
[62]	0.86	0.80	0.50	1.00	1.00	1.00	1.00	**6.16**
[46]	0.86	0.80	0.50	0.83	1.00	1.00	1.00	**5.99**
[63]	0.86	0.80	1.00	0.83	0.00	1.00	0.67	**5.16**
[64]	0.86	0.40	0.50	1.00	0.00	1.00	0.67	**4.42**

**Table 4:** Assessment goals and fatigue measures among studies.

Study	Quality Score	Assessment Goals	Reference Assessment Variables	Non-invasive Physiological Variables (Objective Fatigue Variables)	Subjective Fatigue Assessment	Outcomes
[61]	**6.69**	**Energy expenditure determination**	Equation-based estimates of energy expenditure	Heart rate (resting, mean, %maximal)	None	✓ Equation-based estimates of energy expenditure during heavy-intensity activities were not significantly different from and highly correlated with heart rate-based estimates ✓ There was a small and non-significant bias and good precision between methods ✓ The mean absolute and relative heart rates during the 10, 15 and 20 km loaded runs suggested a physical effort between “vigorous” and “near to maximal” intensity
[45]	**6.46**	**Level of physical exertion (neuromuscular function)**	Vertical jump power, vertical jump height	Maximal heart rate (%), and heart rate reserve (%)	None	✓ Lighter individuals were disadvantaged when carrying absolute loads, as they experienced higher cardiovascular strain (% heart rate reserve) and greater decreases in neuromuscular function (body jump decrease) ✓ Cardiovascular strain corresponded to a “hard” exercise intensity
[59]	**6.36**	**Effects of training**	Biochemical stress markers in blood and saliva (cortisol, dehydroepiandrosterone-sulfate DHEA-s, epinephrine, norepinephrine, soluble transferrin receptors sTfR, neuropeptide-Y NPY, prolactin, testosterone)	Heart rate (mean)	None	✓ When exposed to simulated captivity, stress hormones, heart rate, cognition, mood and nutritional status were simultaneously altered, and each of them subsequently recovered at different rates ✓ Cortisol, DHEA-s, prolactin and testosterone were significantly lower ✓ Epinephrine, norepinephrine and neuropeptide-Y, and heart rate increased ✓ Heart rate increased by 42% and 81% (from baseline) during the two interrogation phases of training
[47]	**6.30**	**Effects of acclimatisation**	Rectal temperature	Heart rate (mean, % theoretical maximal heart rate)	Borg’s RPE scale	✓ Heart rate, thermal discomfort at rest and at the end of the exercise, rates of perceived exertion and sweat loss and osmolality decreased following heat acclimatisation ✓ Decreases in rectal temperature were not significant ✓ Adding short (<60 min) daily moderate-intensity training sessions during mission in a hot and dry environment accelerated heat acclimatisation induced changes at rest and during exercise over 5 days
[48]	**6.30**	**Effects of acclimatisation**	Rectal temperature	Heart rate (resting, mean), facial skin temperature	Modified Borg’s RPE scale	✓ Heat metabolic strain decreased during acclimatisation, as evidenced by the decrease in both rectal temperature and heart rate ✓ Overall, a low-volume training regimen in a hot and dry environment has a modest impact on physiological adaptation ✓ Decreases in facial temperature evolved similarly to thermal discomfort and ratings of perceived exertion in both groups during the heat acclimatisation process
[56]	**6.19**	**Impact of training on injury incidences**	Numbers of injury incidences	Heart rate (mean) and physical activity-related variables	None	✓ Multiple linear regression evidenced that high physical demands, monotony in weekly physical demands, decreasing the development of distances covered on foot, little time for night rest, and little time spent on sport-related physical training were significant risk factors for injuries (together, they described 98.8% of the incidences)
[60]	**6.16**	**Effects of training**	Biochemical stress markers in blood and saliva (cortisol, dehydroepiandrosterone-DHEA)	Sleep, rest and activity periods (min) based on acceleration counts	Multidimensional Fatigue Inventory (MFI)	✓ All the variables were degraded during training but recovered after its completion ✓ Almost all measures were most degraded in the more intense interrogation scenario ✓ The training induced significant but reversible effects on psychological and physiological function—necessary preconditions for stress inoculation training
[62]	**6.16**	**Sex differences in training loads**	NA	Heart rate	Modified Borg’s RPE scale	✓ Daily rate of perceived exertion demonstrated good agreement with heart rate ✓ Women spent more time in the “hard” and “very hard” heart rate zones. However, average daily heart rate values and rates of perceived exertion were not different between sexes
[46]	**5.99**	**Level of physical exertion**	Biochemical stress markers in blood and saliva (testosterone, sex hormone-binding globulin, cortisol and insulin-like growth factor, α-amylase)	Heart rate (absolute, relative, mean, peak), physical activity (metabolic equivalent intensities, step counts)	Borg’s RPE scale	✓ Low quantity of physical activity, low heart rate values, and subjective ratings of exertion proved a “light” physical workload during mission ✓ Stress biomarkers and heart rate responses were proportional and presented no significant changes ✓ No signs of physical overload due to the calm operative nature of the working environment
[53]	**5.82**	**Comparison of physical activity from two training sites**	NA	Accelerometer-based physical activity	None	✓ Recruits from two training sites showed similar amounts of time in physical activity variables, regardless of site and measurement method (accelerometers, physical activity logs and trackers)
[58]	**5.59**	**Heat strain assessment**	Core temperature	Heart rate (mean)	None	✓ Average values reached 140 bpm for heart rate and 39 °C for core temperature ✓ The proposed algorithm had a small bias (0.02 °C) ✓ The overall root mean square error was lower than previous studies comparing different measures of core temperature
[54]	**5.22**	**Heat stress assessment, level of physical exertion**	Core temperature	Heart rate (mean), accelerometry counts	None	✓ Changes in mean heart rate and peak heart rate values proportionally increased during heat strain (when core temperature was >38 °C)
[57]	**5.16**	**Physiological and mental workload**	NA	Heart rate, frequency of breath, skin body temperature, acceleration counts	None	✓ Attrition was predicted by physiological and mental determinants
[63]	**5.16**	**Energy balance during training**	NA	Accelerometer-based physical activity (frequency, length and intensity of physical movement)	None	✓ The average daily number of steps in one week of continuous training was determined as “regular moderately intensive” movement without any competitive sports ✓ During continuous training, a positive energy balance resulted in saving excess energy as fat reserves
[49]	**5.09**	**Heat strain assessment**	Rectal temperature, rate of fluid loss, weight loss	Heart rate (peak), skin temperature (thigh position)	None	✓ Peak heart rate (160 bpm) responded to heat stress reported by rectal temperature (38.4 °C) during patrol activities ✓ Recovery periods did not achieve basal heart rate during the hot exposure ✓ Skin temperature decreased significantly during recovery and rose during exertion
		**Level of physical exertion**	Oxygen consumption	Heart rate (mean)	None	✓ Mean values of heart rate from 140 to 160 bpm were reached during the highly intense patrol activities (3 mL/min) ✓ Mean values of heart rate corresponded to the oxygen consumption during the physical activity
[50]	**4.99**	**Sleep deprivation, physical activity, work–rest cycle, cognitive degradation**	Melatonin	Actigraphy (activity counts, number of sleep periods and time)	None	✓ During the field testing, subjects slept 3.0 ± 0.3 h, with a mean number of sleep intervals of 14.4 ± 1.0 ✓ These results showed a correlation with the low levels of melatonin during the field testing
[51]	**4.99**	**Level of physical exertion in different combat techniques**	NA	Heart rate, accelerometer-based physical activity	None	✓ Differences between symmetrical and asymmetrical combat were evident ✓ Heart rate values during symmetrical and asymmetrical combat conditions were less than 80% of maximal heart rate
[53]	**4.82**	**Comparison of physical activity assessments**	NA	Accelerometer-based physical activity	None	✓ The ActiGraph gave the best measure of the recruits’ physical activity intensity ✓ The physical activity tracker and daily physical activity log were best for body position and type of physical activity ✓ The use of multiple physical activity measurement instruments was necessary to characterise the physical demands of training best
[64]	**4.42**	**Intensity of physical activity**	Maximal oxygen consumption, lactate threshold	Heart rate (mean, % maximal)	None	✓ % of the maximal theoretical heart rate reaching “moderate” to “high” intensity levels during hiking with loads ✓ Outcomes suggest a decrease in work output, early-onset fatigue and increased risk of injury
[55]	**4.09**	**Level of physical exertion**	Oxygen consumption	Heart rate (mean, maximum, maximal)	Borg’s RPE scale	✓ No significant correlations were observed between oxygen consumption and heart rate in the selected military tasks ✓ The significant increase in heart rate is not related to respective oxygen consumption values measured during the last hour of loaded marching

## Data Availability

Not applicable.

## Appendix A

**Table A1:** Risk of bias assessment criteria.

Categories	No.	Assessment Questions	Selected Studies																			
			[64]	[57]	[58]	[47]	[51]	[61]	[59]	[48]	[62]	[46]	[52]	[53]	[60]	[56]	[63]	[54]	[55]	[49]	[45]	[50]
Study design	1	Were the aims and objectives defined clearly?	Y	Y	Y	Y	Y	Y	Y	Y	Y	Y	Y	Y	Y	Y	Y	Y	Y	Y	Y	Y
	2	Were the aims and design of the study set in the context of existing knowledge?	Y	Y	Y	Y	Y	Y	Y	Y	Y	Y	Y	Y	Y	Y	Y	Y	Y	Y	Y	Y
	3	Were the outcome measures defined clearly?	Y	Y	Y	Y	Y	Y	Y	Y	Y	Y	Y	Y	Y	Y	Y	Y	Y	Y	Y	Y
	4	Was there a control group?	N	N	N	Y	N	N	N	Y	N	N	N	N	N	N	N	N	N	N	N	N
	5	Was the research design suitable to answer the research question?	Y	Y	Y	Y	Y	Y	Y	Y	Y	Y	Y	Y	Y	Y	Y	Y	Y	Y	Y	Y
	6	Was there a clear description of the applied methods and equipment?	Y	Y	Y	Y	Y	Y	Y	Y	Y	Y	Y	Y	Y	Y	Y	Y	Y	Y	Y	Y
	7	Were the methods of statistical analysis described?	Y	Y	Y	Y	Y	Y	Y	Y	Y	Y	Y	Y	Y	Y	Y	Y	Y	Y	Y	Y
Participants	8	Were ethical standards met? (including informed consent and ethical approval)	Y	Y	Y	Y	Y	Y	Y	Y	Y	Y	Y	Y	Y	Y	Y	U	Y	Y	Y	Y
	9	Was the sample selection adequate and justified? (e.g., power calculation)	N	Y	N	Y	Y	Y	Y	Y	Y	Y	Y	Y	Y	Y	Y	N	N	N	Y	Y
	10	Was a method of randomisation performed?	N	N	N	N	N	Y	Y	N	N	N	N	N	N	Y	N	N	N	N	N	N
	11	Were the groups similar at baseline regarding the most important prognostic indicators?	Y	Y	Y	Y	Y	Y	Y	Y	Y	Y	Y	Y	Y	Y	Y	Y	Y	Y	Y	Y
	12	Was the number of subjects representative to assure the statistical power of the study?	N	Y	N	Y	Y	Y	Y	Y	Y	Y	Y	Y	Y	Y	Y	N	N	N	N	Y
Data sources	13	Was the study developed in a regular operational or training military context?	Y	Y	Y	Y	Y	Y	Y	Y	Y	Y	Y	Y	Y	Y	Y	Y	Y	Y	Y	Y
	14	Did all subjects complete all testing?	N	N	N	N	U	Y	N	N	N	N	N	N	N	N	Y	N	N	Y	Y	N
Reporting bias	15	Were the sample characteristics adequately provided?	Y	Y	Y	Y	Y	Y	Y	Y	Y	Y	N	N	Y	Y	Y	Y	Y	Y	Y	Y
	16	Was there a description of withdrawals and dropouts?	Y	Y	N	Y	N	U	Y	Y	Y	N	N	N	Y	N	N	Y	Y	U	Y	N
	17	Were statements and numerical data supported by the literature’s citations?	Y	Y	Y	Y	Y	Y	Y	Y	Y	Y	Y	Y	Y	Y	Y	Y	Y	Y	Y	Y
	18	Did the study provide complete outcome data for each main outcome, including attrition and exclusions from the analysis?	Y	Y	Y	Y	Y	Y	Y	Y	Y	Y	Y	Y	Y	Y	Y	Y	Y	Y	Y	Y
	19	Were tables, figures and retrospective analyses presented clearly?	Y	Y	Y	Y	Y	Y	Y	Y	Y	Y	Y	Y	Y	Y	Y	Y	Y	Y	Y	Y
	20	Did the data support the authors’ conclusions?	Y	Y	Y	Y	Y	Y	Y	Y	Y	Y	Y	Y	Y	Y	Y	Y	Y	Y	Y	Y
Limitations	21	Were the limitations of the study objectively defined?	N	N	Y	Y	N	Y	Y	Y	Y	Y	N	Y	Y	Y	N	Y	Y	N	Y	N
Generalisability	22	Does the study allow generalisation? Did data lead to conclusive results?	Y	Y	Y	Y	Y	Y	Y	Y	Y	Y	Y	Y	Y	Y	Y	Y	N	Y	Y	Y
Potential sources of bias	23	Was the withdrawal/drop-out rate unlikely to cause bias?	Y	Y	Y	Y	Y	Y	Y	Y	Y	Y	Y	Y	Y	Y	Y	Y	N	Y	Y	Y
	24	Was the number of missing values unable to compromise a meaningful analysis?	N	Y	Y	Y	Y	Y	Y	Y	Y	Y	Y	Y	Y	Y	Y	Y	N	Y	Y	Y
	25	Were practical difficulties (e.g., in recruitment or loss to follow-up) unable to lead to major compromises in study implementation compared with the study protocol?	Y	Y	Y	Y	Y	Y	Y	Y	Y	Y	Y	Y	Y	Y	U	N	Y	Y	Y	Y

Y: yes; N: no; U: unclear.

## Appendix B

**Table A2:** Summary of manuscripts assessing heart rate-derived variables.

Study	Control Group or Basal Comparison	Context	Heart Rate–Derived Variables	Equipment
[49]	Basal comparison	Military field exercises	Mean heart rate (bpm), peak heart rate (bpm), maximum heart rate (bpm)	Polar Sport tester heart rate monitors at 1 min intervals
[45]	Basal comparison	Recruit training course	Mean heart rate (bpm), resting heart rate (bpm), maximum heart rate (bpm), maximal heart rate (%), heart rate reserve (%)	Polar Team System Polar, Kempele, Finland
[51]	Basal comparison	Course of physical education instructors of the army	Mean heart rate (bpm), maximal heart rate (%)	Heart rate transmitter belt Polar Electro, Kempele, Finland
[54]	Basal comparison	Three dismounted missions	Mean heart rate (bpm)	Equivital EQ-01 Hidalgo, Cambridge, United Kingdom (15 s intervals)
[55]	Basal comparison	Military field exercises	Maximal heart rate (%), mean heart rate (bpm), maximum heart rate (bpm)	Lab: Polar S610, Polar Electro, Kempele, Finland Military tasks: Suunto t6, Suunto, Finland
[56]	Basal comparison	Army basic military training	Mean heart rate (bpm)	Heart rate monitors: Suunto Smartbelt and Comfortbelt; Suunto, Vantaa, Finland
[57]	Basal comparison	Marine initial training course	Mean heart rate (bpm), heart rate reserve (bpm)	EquivitalTM EQ02 Life Monitor; Hidalgo Limited, Cambridge, UK
[58]	Basal comparison	Three CBRNE training events	Mean heart rate (bpm)	Equivital EQ02, Hidalgo Ltd., Cambridge, UK (15 s intervals)
[59]	Basal comparison	Captivity survival training	Mean heart rate (bpm)	EquiVital type 1 Sensor Electronics Module Hidalgo LTD., Cambridge, U.K. (256 samples per second)
[47]	Control group	Aerobic training/regular military operations	Mean heart rate (bpm), theoretical maximal heart rate (%)	Monitor wrist receptor RC3 GPS, Polar, Kempele, Finland
[61]	Basal comparison	Army Ranger training courses	Resting heart rate (bpm), mean heart rate (bpm), maximal heart rate (%)	Memory belt, Suunto, Finland, (acquisition frequency: 10 s)
[48]	Control group	Aerobic training programme/usual outdoor military activities	Resting heart rate (bpm), mean heart rate (bpm)	Polar RC3 GPS, Kempele, Finland
[62]	Basal comparison	Standard entry basic military training	Heart rate reserve (%), minimal and maximal heart rate (bpm), mean heart rate (bpm)	Heart rate monitor Polar Team Pro, Polar, Finland
[46]	Basal comparison	International crisis management operation	Absolute and relative mean heart rate (bpm), minimum and peak heart rate (bpm)	Memory belt, Suunto, Vantaa, Finland
[64]	Basal comparison	Laboratory testing/MWCW training	Mean heart rate (bpm), maximal heart rate (bpm), maximal heart rate (%), peak heart rate (bpm)	Heart rate monitor Polar USA, Lake Success, NY (in 5 s epochs)

CBRNE = Chemical, biological, radiological, nuclear, or explosive agents training. MWCW = Mountain warfare/cold weather training.

**Table A3:** Summary of manuscripts assessing physical activity variables.

Study	Control Group or Basal Comparison	Context	Physical Activity Variables	Equipment
[50]	Basal comparison	Combat training	Activity counts estimated duration and fragmentation of sleep (h)	Motionlogger Actigraphs, model BMA-32; Precision Control Devices, Ft. Walton Beach, Florida
[51]	Basal comparison	Course of physical education instructors of the army	Acceleration counts, velocity, distance, position, direction, number and intensity of impacts	Triaxial built-in accelerometer with an operational sampling rate of 100 Hz; GPS
[52]	Basal comparison	Basic combat training	Acceleration counts, average daily time (min) in sedentary, light-, moderate- and vigorous-intensity activities, time asleep vs. time awake, time in each body position, time in each type of physical activity	ActiGraph GT3X triaxial accelerometer, Pensacola, FL. PAtracker. Army-developed direct observation tool
[53]	Basal comparison	Basic combat training	Acceleration counts, average daily time (min) in sedentary, light-, moderate- and vigorous-intensity activities, time asleep vs. time awake, time in each body position, time in each type of physical activity	ActiGraph GT3X triaxial accelerometer, Pensacola, FL. PAtracker. Army-developed direct observation tool
[54]	Basal comparison	Three dismounted missions	Accelerometry counts	Equivital EQ-01 (Hidalgo, Cambridge, United Kingdom)
[56]	Basal comparison	Army basic military training	Hip acceleration counts, step counts, backpack acceleration data	GT1M (ActiGraph, Fort Walton Beach, Florida) and PARTwear (HuCE microLab, Biel, Switzerland) (at 2 s intervals)
[57]	Basal comparison	Marine initial training course	Acceleration counts	EquivitalTM EQ02 Life Monitor; Hidalgo Limited, Cambridge, United Kingdom
[46]	Basal comparison	International crisis management operation	Metabolic equivalent intensities (MET), step counts	Tri-axial accelerometer (at a frequency of 100 Hz) Hookie AM20, Traxmeet, Espoo, Finland
[60]	Basal comparison	Captivity survival training	Sleep, rest and activity periods (min) based on acceleration counts	Wrist actigraph Ambulatory Monitoring, Inc., Ardsley, NY, USA
[63]	Basal comparison	Military training	Acceleration counts, length (m) and intensity of physical movement (METs)	ActiGraph GT1M (1 min intervals)
